# The Prevalence of Anxiety Among Children in Saudi Arabia During the COVID-19 Pandemic: A Cross-Sectional Study

**DOI:** 10.7759/cureus.48942

**Published:** 2023-11-17

**Authors:** Moudi M Alasmari, Hatoon Alshaikh, Solaf H Alotaibi, Yara Batwa, Mona Y Alsheikh, Heba Alshaeri, Abdulnasser Helali

**Affiliations:** 1 Department of Pharmacology, College of Medicine, King Saud bin Abdulaziz University for Health Sciences (KSAU-HS), Jeddah, SAU; 2 Department of Medicine, College of Medicine, King Saud bin Abdulaziz University for Health Sciences (KSAU-HS), Jeddah, SAU; 3 Department of Clinical Pharmacy, College of Pharmacy, Taif University, Taif, SAU; 4 Department of Pharmaceutical Sciences, Fakeeh College for Medical Sciences, Jeddah, SAU; 5 Department of Pediatrics, King Abdulaziz Medical City (KAMC) Ministry of National Guard - Health Affairs (NGHA), Jeddah, SAU

**Keywords:** childhood anxiety disorders, mental health, children, anxiety, prevalence

## Abstract

Background: The year 2021 was a year that can be perceived as a stressful event given the considerable lifestyle changes that have occurred worldwide due to the pandemic. Several studies have investigated the psychological impact on people during this time period. However, only a few of them have investigated the impact on young children in Saudi Arabia (SA) specifically. Therefore, this study aimed to investigate the prevalence of anxiety in children living in Jeddah, Saudi Arabia.

Methods: This is a cross-sectional study that included the parents of 388 children aged 6-9 years living in Jeddah, Saudi Arabia. It was conducted from June to November 2021. The parents completed an electronic survey that included the Arabic version of the Spence Children’s Anxiety Scale. The data were analyzed using John’s Macintosh Project (JMP) software version 10.0 (SAS Institute Inc., Cary, NC).

Results: A total of 388 responses were collected. The data revealed that 37 (9.5%) participants experienced anxiety. Furthermore, the prevalence of anxiety was higher among females (5.15%) as compared to males (4.38%), but the difference was not statistically significant.

Conclusion: These findings highlight the importance of recognizing the psychological burden in young populations and emphasize the importance of taking children’s well-being into account.

## Introduction

Anxiety is when an individual’s thoughts are filled with fear and worry [1]. According to the fifth edition of the Diagnostic and Statistical Manual of Mental Disorders (DSM-5), anxiety disorders include several types, such as generalized anxiety disorder, panic disorder, social anxiety, and a number of other disorders including phobia-related disorders [2]. Many children and young people experience anxiety as a normal part of life, just like adults; however, when it is intense and persistent, it can be a sign of a mood disorder [3]. In childhood, the risk of anxiety disorder is higher; thus, early recognition and assessment, as well as determination of the presence of this disorder, is necessary for controlling anxiety and its consequences in later life [4]. Anxiety disorder has a long-lasting negative impact on different aspects of a person’s life; it affects cognitive, behavioral, and social functions in different stages of life, which can manifest as low self-esteem, depression, or even educational failure [5].

According to the 2017 global health estimate of the World Health Organization (WHO) for common mental disorders, there were 264 million individuals around the globe who were affected by various types of anxiety disorders, mostly females [6]. In Saudi Arabia (SA), a study reported that the prevalence of childhood anxiety disorder among primary school children in 2019 was 42% [7]. Another study that investigated generalized anxiety disorder (GAD) prevalence in 2020 among youth in different regions of SA showed that 78.9% of the participants had shown symptoms of GAD [8]. These numbers signal the depth of anxiety problems globally as well as locally as it is a common and growing mental health issue.

Information on anxiety among children in SA during recent years is still insufficient, and as of the time this study was conducted, there are no available studies investigating the prevalence of anxiety in children during the year 2021 in Jeddah, Saudi Arabia. Additionally, considering the remarkable spike in the number of adverse psychological events in the general population in the year 2020-2021 due to the contributing pressure of the COVID-19 pandemic and its role in drastic lifestyle and quality of life changes, as well as the scant studies concerned with children’s mental health generally and anxiety specifically during the pandemic, this study aimed to estimate the prevalence of anxiety in children in 2021 in Jeddah, Saudi Arabia, and to raise awareness of children’s psychological needs, thereby drawing attention to the importance of detecting early signs of anxiety and negative emotions in children and providing the needed care for their emotional and overall well-being in the future.

## Materials and methods

Study design and participants

A cross-sectional study was conducted to assess the prevalence of children’s anxiety during the year 2021 in Jeddah, Saudi Arabia, from June to November 2021. The survey used included the Arabic version of the Spence Children’s Anxiety Scale, which is validated for children 6-9 years of age. The survey was electronically distributed through different social media platforms, such as WhatsApp and Telegram. Convenience sampling and the snowball method were used.

Sample size

The population of children from ages 6-9 in Jeddah, Saudi Arabia, was estimated to be around 350,000 in 2020 [9]. The sample size was calculated using Raosoft® software, with a 5% margin of error, a confidence level of 95%, and an estimated 50% response distribution; the determined sample size was 384.

Questionnaire

The survey contained two sections, and these included both multiple-choice and dichotomous questions. The first section of the survey was designed to collect demographic information from the participants, including the age of the child, gender, nationality, having siblings, and the marital status of the parents. The second section of the survey included questions from the Arabic version of Spence Children’s Anxiety Scale (Parent Version), which was validated only for children aged 6-9 years. It is a 4-point Likert scale that includes 39 questions that investigate anxiety symptoms in children. The scale also includes six subscales that are designed to detect the symptoms of fear of physical injury, social phobia, separation anxiety, generalized anxiety/overanxious symptoms, obsessive-compulsive problems, and panic/agoraphobia. The maximum scores for the subscales are 18 for obsessive-compulsive problems, generalized anxiety, separation anxiety, and social phobia; 15 for fear of physical injury; and 27 for panic/agoraphobia. To measure general anxiety, the points obtained from the scale’s questions are added, and the totals are compared to equivalent calculated T-scores. If a child has a total that is equivalent to a T-score of 60 or more, this indicates that the child has anxiety symptoms. Furthermore, the reliability and validity of the scale were proven after a psychometric study, and the Cronbach’s alpha coefficient (internal consistency) of the scale was 0.73 [10].

Ethical considerations

The study ensured complete confidentiality and privacy because no identifiers were collected and all data, both hard and soft copies, were saved within the Ministry of National Guard - Health Affairs (MNGHA) and King Abdullah International Medical Research Center (KAIMRC) premises and were accessible only to the research team. Institutional review board (IRB) approval was obtained (reference number: JED-21-427780-38967).

Data analysis

Data was entered into Microsoft Excel (Microsoft Corp., Redmond, WA) and analyzed using the John’s Macintosh Project (JMP) software version 10.0 (SAS Institute Inc., Cary, NC). Numerical variables, such as age, were not normally distributed and were presented as median, maximum, and minimum values, and categorical variables, such as gender, marital status, nationality, and having siblings (yes/no), were presented as frequencies (%). Furthermore, the prevalence of anxiety for all the respondents who scored 60 or more on the scale was found. The association between anxiety symptoms and children’s demographics was investigated using an unpaired t-test and one-way analysis of variance (ANOVA). The association between the average subscale scores and gender was investigated using a nonparametric statistical Wilcoxon test. Findings with a p-value < 0.05 were considered significant.

## Results

Participants’ demographics

Parents of 388 children (196 (51%) males and 192 (49%) females) completed the parents’ version of the Spence Children’s Anxiety Scale. The distribution of the children’s ages was as follows: six years, 28%; seven years, 20%; eight years, 19%; and nine years, 33%. The data on the parents’ marital status were as follows: married, 91%; divorced, 6%; and widowed, 3%. Moreover, 89% of the children were Saudis, while 11% were non-Saudis. Finally, 95% of the children had siblings, while just 5% were only children (Table 1).

**Table 1:** Profile of the study participants

Demographic variables	Frequency	Percentage (%)
Age (years)		
6	107	28
7	79	20
8	73	19
9	129	33
Gender		
Male	196	51
Female	192	49
Parents’ marital status		
Married	355	91
Divorced	23	6
Widowed	10	3
Nationality		
Saudi	347	89
Non-Saudi	41	11
Siblings		
Yes	368	95
No	20	5

Prevalence of anxiety

A total of 37 (9.5%) children had anxiety symptoms; 17 of them were male, and 20 were female. Ten (2.57 %) of the children were six years, five (1.28%) were seven years, six (1.54%) were eight years, and 16 (4.12%) were nine years. Regarding the parents’ marital status, 34 (8.76%) were married, two (0.52%) were widowed, and only one (0.26%) was divorced. Thirty-two (8.2%) of the children with anxiety were Saudis, while only five (1.3%) were non-Saudis. Only three children did not have siblings (Table 2). There was no significant association between the prevalence of total anxiety and any of the children’s demographics.

**Table 2:** Demographics of children with anxiety

Demographic variables	Anxiety		p-value
	Yes	No	
Age (years)			
6	10 (2.58%)	97 (25%)	0.50
7	5 (1.29%)	74 (19.07%)	
8	6 (1.55%)	67 (17.27%)	
9	16 (4.12%)	113 (29.12%)	
Gender			
Male	17 (4.38%)	179 (46.13%)	0.13
Female	20 (5.15%)	172 (44.33%)	
Parents’ marital status			
Married	34 (8.76%)	321 (82.73%)	0.37
Divorced	1 (0.26%)	22 (5.67%)	
Widowed	2 (0.52%)	8 (2.06%)	
Nationality			
Saudi	32 (8.2%)	315 (81.19%)	0.53
Non-Saudi	5 (1.3%)	36 (9.28%)	
Siblings			
Yes	34 (8.76%)	334 (86.08%)	0.39
No	3 (0.77%)	17 (4.38%)	

Associations between the subscales and gender

The median, maximum, and minimum values for each of the six subscales were found for both genders, as they were not normally distributed. The median values of the social phobia and fear of physical injury scores were higher in females. When examining the association between fear of physical injury and gender, it was found that the median was higher in females, with a significant p-value of 0.002. Similarly, regarding social phobia, it was found that the median was also higher in females, with a significant p-value of 0.003. As for the remaining subscales, panic/agoraphobia, separation anxiety, generalized anxiety, and OCD, no significant association with gender was found (Table 3).

**Table 3:** Association between the scoring results of the subscales and gender *Statistically significant

Anxiety subscales	Males’ scale score median (minimum-maximum)	Females’ scale score median (minimum-maximum)	p-value
Generalized anxiety disorder	4 (0-15)	4 (0-16)	0.81
Separation anxiety	6 (0-16)	7 (0-18)	0.49
Social phobia	4 (0-14)	5 (0-16)	0.003*
Panic/agoraphobia	2 (0-18)	2 (0-23)	0.40
Obsessive-compulsive problems	2 (0-9)	2 (0-15)	0.64
Fear of physical injury	5 (0-13)	6 (0-15)	0.002*

## Discussion

Considering the remarkable lifestyle changes that occurred worldwide during the COVID-19 pandemic and the fear it caused, several studies have investigated the psychological impact of this time period. According to the literature search conducted, there are very few studies on the prevalence of mental health issues in children during the period of COVID-19 pandemic in Saudi Arabia. Moreover, to our knowledge, this cross-sectional study is the first to reveal the prevalence of anxiety in children during the pandemic period of 2021 in Jeddah, Saudi Arabia. Based on our findings, the prevalence of such was around 9.5%.

The results indicate that the prevalence of anxiety symptoms (n=37) was higher in female children as 20 (5.15%) of the 37 children who experienced anxiety were females and 17 (4.38%) were males, although the difference was not statistically significant. Also, when examining the association between gender and the subscales, females showed significantly higher average scores for fear of physical injury and social phobia. Furthermore, there was no significant association between any of the children’s demographics and anxiety.

Locally, according to a study reported by Alfakeh et al. [7], the prevalence of anxiety in children aged 6-12 in Saudi Arabia in 2019 was 42.1%, which is a higher percentage than the percentage found in our study; however, this discrepancy could be explained by the fact that the previous study included a wider age range and a larger sample size. Additionally, one of our findings was that the prevalence of anxiety was higher among females, which is in agreement with a number of previous studies. For example, a global systemic review performed in 2021 that included 29 studies from 10 countries examined the prevalence of anxiety and depression in children and adolescents and found that females had higher rates of anxiety as compared to males [11]. Moreover, another similar meta-analysis, which included 23 studies from China and Turkey, also showed higher rates of anxiety in female children as compared to males [12]. The exact explanation for the difference in gender is unclear; however, one study suggested that having lower self-esteem, biological susceptibility, and a higher probability of experiencing interpersonal violence are all factors that could lead to a higher prevalence of anxiety in females [11].

Generally, phobias are found to have a higher prevalence among females as compared to males [13]. The findings of this study showed that females have higher average scores for both fear of physical injury and social phobia. Other studies have also shown the same findings. For instance, according to a study that investigated social phobia symptoms and separation anxiety symptoms in children and adolescents, females reported higher levels of social phobia than males [14]. Moreover, another study on anxiety symptoms in Japanese and German children reported that females had higher social phobia symptoms and fear of physical injury as compared to males [15]. Furthermore, one study conducted in the province of Alicante in Spain on anxiety disorder symptoms in children and adolescents also reported that females displayed higher levels of social phobia and physical fear than males [16].

Due to the methodological choices of this study, there are some limitations, one of which is the small age group because the Arabic version of the survey was only validated for children aged 6-9 years. Also, the survey was filled out by the parents, which can impact the results as the parents may provide subjective interpretations of their child’s behavior.

## Conclusions

Around 10% of our sample had symptoms of anxiety. Conducting this type of epidemiological research is crucial to recognizing the psychological burden in young populations because of drastic global lifestyle changes. The results of this study emphasize the importance of taking children’s well-being into account and continuously enhancing the psychosocial support provided to them during such difficult circumstances. Further studies with a larger sample size, age range, and regional area are recommended. Moreover, factors that contribute to anxiety, such as a family’s socioeconomic status and whether the children are on certain medications or have other general medical conditions, should also be taken into account.
